# Charting the transcriptional landscape of cells of renin lineage following podocyte depletion

**DOI:** 10.1371/journal.pone.0189084

**Published:** 2017-12-12

**Authors:** Aaron D. McClelland, Julia Lichtnekert, Diana G. Eng, Jeffrey W. Pippin, Kenneth W. Gross, Sina A. Gharib, Stuart J. Shankland

**Affiliations:** 1 Division of Nephrology, University of Washington, Seattle, Washington, United States of America; 2 Department of Molecular and Cellular Biology, Roswell Park Cancer Institute, Buffalo, New York, United States of America; 3 Computational Medicine Core, Center for Lung Biology, Division of Pulmonary, Critical Care and Sleep Medicine, University of Washington, Seattle, Washington, United States of America; Temple University, UNITED STATES

## Abstract

Renin producing cells of the juxtaglomerulus, herein called cells of renin lineage (CoRL), have garnered recent interest for their propensity to act as a progenitor source for various kidney cell types including podocytes. Despite recent advances, the process of transdifferentiation of CoRL to podocytes is poorly understood. In this study, we employed a transgenic reporter mouse line which permanently labels CoRL with ZsGreen fluorescent protein, allowing for isolation by fluorescence-activated cell sorting. At 5 days following induction of abrupt podocyte ablation via anti-podocyte sheep IgG, mice were sacrificed and CoRL were isolated by FACS. RNA was subsequently analyzed by microarray. Gene set enrichment analysis (GSEA) was performed and revealed that CoRL display a distinct phenotype following podocyte ablation, primarily consisting of downregulation of metabolic processes and upregulation of immuno-modulatory processes. Additionally, RNA-biology and cell cycle-related processes were also upregulated. Changes in gene expression or activity of a core set of transcription factors including HNF1 and E2F were identified through changes in enrichment of their respective target genes. However, integration of results from *transcription factor* and *canonical pathway* analysis indicated that ERR1 and PU-box family members may be the major contributors to the post-podocyte ablation phenotype of CoRL. Finally, top ranking genes were selected from the microarray-based analysis and confirmed by qPCR. Collectively, our results provide valuable insights into the transcriptional regulation of CoRL following abrupt podocyte ablation.

## Introduction

The kidney is a highly specialized and structurally complex organ comprised of several unique cell types, each of which serve distinct roles in metabolite reclamation and urine concentration [1–4]. Additionally, the kidney is pivotal in systemic blood pressure regulation through its near exclusive production of renin, the rate limiting enzyme in the renin-angiotensin system. Kidney renin secretion is mediated by a small population of cells that associate with the afferent glomerular arteriole, which in conjunction with the arteriole endothelial cells and smooth muscle cells comprise the juxtaglomerular apparatus [5]. This structure forms an integral link between upstream and downstream sections of the nephron via the macula densa in the distal proximal tubule resulting in so-called tubulo-glomerular feedback [6].

Recently, these renin secreting juxtaglomerular cells, herein called cells of renin lineage (CoRL), have been demonstrated to act as a progenitor reservoir for various cells within the glomerulus for both epithelial and mesenchymal lineages [7, 8]. In particular, lineage tracing experiments has shown that following podocyte loss in experimental disease, a subset of CoRL migrate from the afferent arteriole into the glomerular tuft, where they *de novo* express several podocyte markers, and a subset exhibit ultrastructural features of podocytes [9]. Importantly, translocation and differentiation of CoRL on the tuft occurs concomitantly with stabilization or in some cases, decreases in albumin secretion, along with increase in podocyte density. Administration of ACE-inhibitors and angiotensin receptor blockers augment this reparative phenotype thereby supporting this novel paradigm of *de novo* post-natal podocyte regeneration [10].

This emergent field has important clinical implications especially in the various nephropathies typified by podocyte depletion such as diabetic kidney disease, membranous nephropathy and many forms of FSGS [11]. However, the molecular pathways activated and suppressed in CoRL under disease states remain poorly understood. Using CoRL reporter mice which enabled FACS sorting of labeled CoRL, we employed an experimental model of FSGS to determine the transcriptional consequences of abrupt podocyte loss in this cell population.

## Materials and methods

### Study animals

Ren1cCre×Rs-ZsGreen-R reporter mouse labels the Ren1c expressing cells with ZsGreen. These are derived from *Ren1c*Cre mice which contain Cre recombinase under control of the renin regulatory region [12] and the B6.Cg-*Gt(ROSA)26Sor*^*tm6(CAG-ZsGreen1)Hze*^/J which contain a *loxP*-flanked STOP cassette controlling CAG-driven expression of ZsGreen (Jackson Labs Stock # 007906) [13]. Thus, progeny containing the *Ren1c*Cre transgene in addition to the *loxP*-controlled ZsGreen cassette, constitutively and permanently label any cells which express, or have expressed renin.

### Isolation of ZsGreen labeled CoRL

Fresh mouse kidneys were removed, and the capsules and fat were dissected away under aseptic conditions. Kidney cortex was removed from the rest of the kidney with a sterile scalpel and minced. Tissue was digested in 0.2mg/ml Liberase TL (Sigma-Aldrich, St. Louis, MO), 100 U/ml DNAse I (Sigma-Aldrich, St. Louis, MO) in RPMI 1640 medium, without L-glutamine or phenol red (GE Healthcare Bio-Sciences, Pittsburgh, PA) by shaking in a 37°C water bath for 30 minutes. The digest was passed through a 22G needle (Becton Dickenson, Franklin Lakes, NJ) 10 times to further dissociate the tissue, then inactivated by combining with 5ml of in media consisting of RPMI 1640 medium, without L-glutamine or phenol red (GE Healthcare Bio-Sciences, Pittsburgh, PA) supplemented with 1mM sodium pyruvate (ThermoFisher Scientific, Waltham, MA), 9% Nu-Serum^™^ IV Growth Medium Supplement (Corning Incorporated—Life Sciences, Durham, NC) and 100U/ml Penicillin-Streptomycin (ThermoFisher Scientific, Waltham, MA). The suspension was passed through a 100μm, then a 40 μm cell strainer (BD Biosciences, San Jose, CA), to clear multicellular debris, then pelleted by centrifugation at 200G at 4°C for 5 minutes. The cells were re-suspended in the media described above, counted and isolated using multicolor fluorescence-activated cell sorting (FACS) on a BD FACS Aria II (BD Biosciences, San Jose, CA) housed within a BSL1/2 approved biosafety cabinet. Sorted cells were pelleted by centrifugation and snap frozen in liquid nitrogen until isolation of RNA. The entire procedure took 6-7h per animal.

### Experimental FSGS

Experimental FSGS was induced in Ren1cCre×Rs-ZsGreen-R mice with a cytotoxic anti-glomerular antibody, as we have previously reported [14]. Briefly, two doses of sheep anti-glomerular antibody at 12 mg/20g of body weight via intraperitoneal injection, 24 hours apart, induced abrupt podocyte depletion. Mice were euthanized 5 days following the second dose of sheep anti-glomerular antibody (n = 4). Age matched mice which did not receive sheep anti-glomerular antibody were used as baseline controls (n = 4).

Mice were bred and housed in the animal care facility at the University of Washington under specific pathogen-free conditions and provided ad libitum food and water. Animals euthanized for studies were given a ketamine-xylazine cocktail overdose, prior to cardiac perfusion and tissue harvest. All studies were reviewed and approved by the University of Washington under IACUC protocol (2968–04).

### Microarray pipeline

RNA from FACS isolated ZsGreen CoRL was isolated using Qiagen RNeasy Plus Mini Kit according to the manufacturers protocol. Extracted RNA yield and purity were determined using a NanoDrop ND-1000. RNA integrity was determined using an Agilent 2100 Bioanalyzer, samples with RIN ≥ 8 were considered to be of sufficient quality for microarray analysis. Due to low sample yield, samples were amplified prior to cDNA synthesis and labeling using Ovation Pico WTA System V2 Kit (Nugen, San Carlos, CA). Sample labeling and hybridization was performed using the MouseRef-8 (v2.0) Expression BeadChip Kit (Illumina, San Diego, CA). Microarray image processing and data acquisition was performed using the Illumina iScan system at Fred Hutchinson Cancer Research Center’s Genomics Shared Resource.

Detailed microarray experiment description, meeting Minimum Information About a Microarray Experiment (MIAME) requirements, has been deposited at Gene Expression Omnibus (www.ncbi.nlm.nih.gov/geo, GSE104416).

### Microarray data analysis

Microarray data was assessed for quality and underwent quantile normalization using the Bioconductor package “*lumi*” [15]. Multidimensional scaling using Principal Component Analysis (PCA) was performed based on the entire transcriptional profile of all samples. Differentially expressed genes between baseline control (n = 4) and experimental FSGS samples (n = 4) was determined using a Bayesian implementation of the *t*-test (CyberT, http://cybert.ics.uci.edu/) on log_2_-transformed intensities [16]. Multiple hypothesis testing was addressed using Benjamini-Hochberg’s FDR procedure [17]. Functional enrichment of differentially expressed genes (defined as those with FDR ≤ 0.01) was performed using Database for Annotation, Visualization and Integrated Discovery (DAVID, v6.8) based on Gene Ontology annotations. We used an FDR ≤ 0.01 to designate significant enrichment for a given GO category.

### Gene set enrichment analysis

Following normalization and statistical analysis of the microarray data, probes aligning to the same genomic transcript were collapsed by averaging their data, thereby reducing the number of probe identifiers from 25,698 to 18,138 unique transcripts. The unique gene expression dataset was analyzed using GSEA with 1000 gene set permutations and FDR threshold ≤ 0.01 used for determining significance [18]. The following databases from MsigDB (v6.0) were utilized: Hallmark (50 gene sets), Curated Gene Sets (CP set only, 1329 gene sets), Motif Gene Sets (TFT set only, 615 gene sets) (http://software.broadinstitute.org/gsea/msigdb/collections.jsp) via the javaGSEA desktop application. Relationships among enriched gene sets (FDR ≤ 0.01) were depicted based on the number of shared genes between pathways using a network-based visualization method (www.baderlab.org/Software/EnrichmentMap/) within the Cytoscape environment [19, 20]. Integration of transcription factor and canonical pathway gene set enrichment analysis was performed using R statistical programming language [21]. R packages utilized include *colormap* [22], *gplots* [23], *openxlsx* [24] and *venneuler* [25].

### Circos plot

Circos plots were generated from command line using the *tableviewer* plugin. Following integration of the *transcription factor* and *canonical pathway/hallmark* test results data were converted into a contingency table for use with Circos. Transcription factors which only have targets in a single pathway were not plotted in both baseline and day 5 plots. In baseline samples, only genes, pathways and transcription factors which were significantly dysregulated at FDR ≤ 0.01 were considered. In day 5 samples, only genes, transcription factors and pathways that were significantly dysregulated at FDR ≤ 0.001 were considered. To further reduce plot complexity from day 5 data, only transcription factor-pathway interactions containing at least 4 member genes were plotted.

### Statistical analysis

Statistical analyses were performed using GraphPad Prism 6. Statistical significance was determined with the use of unpaired Student’s *t*-test utilizing Welch’s correction. This accounts for possible inequalities in sample variances between the test groups where appropriate. The significance threshold was set at *P*-value ≤ 0.05 and comparisons exceeding this were deemed non-significant.

## Results

### Isolation of ZsGreen-CoRL RNA from mouse renal cortex

**Fig 1:**
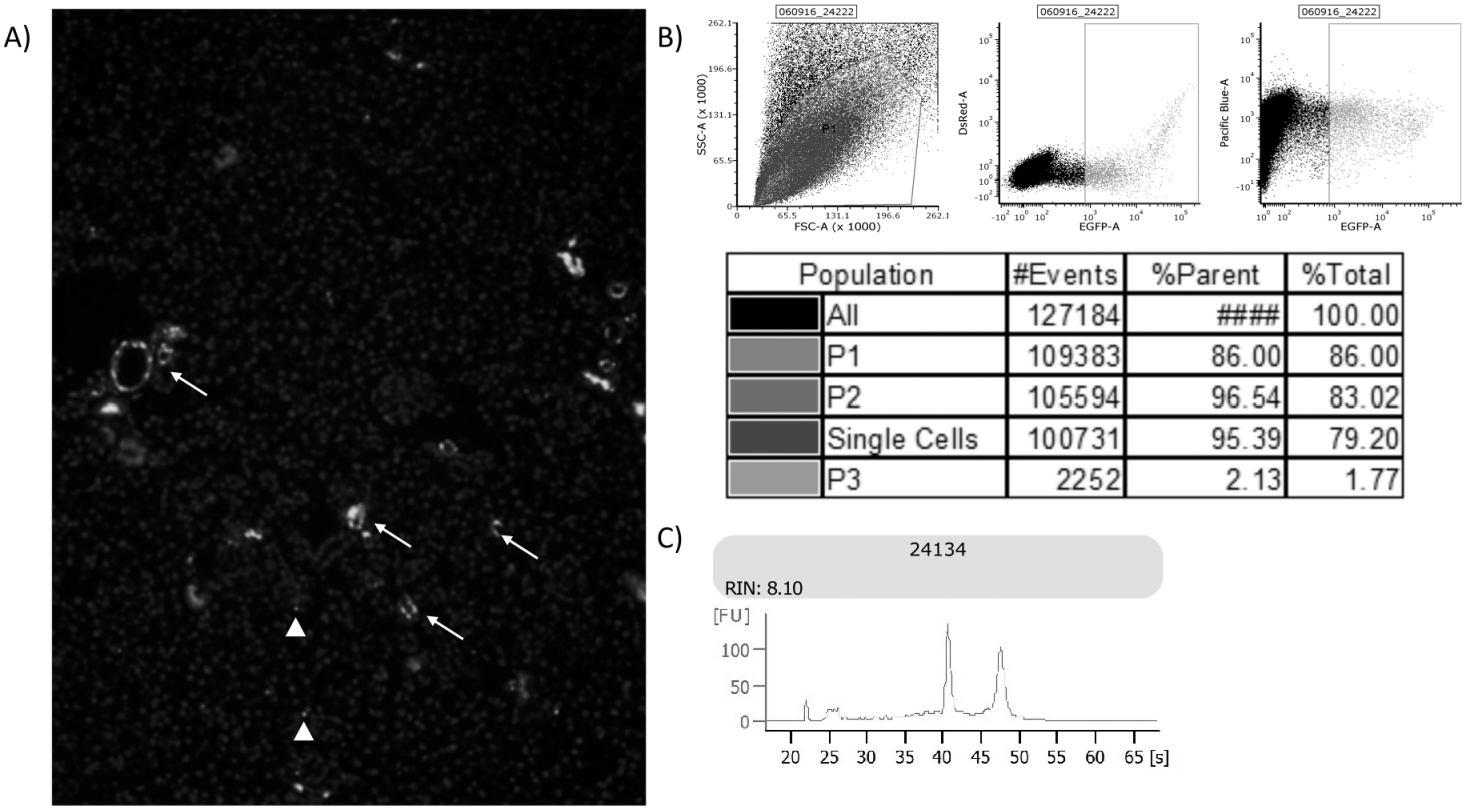
Labeling and isolation of CoRL by FACS. (A) ZsGreen is expressed in juxtaglomerular apparati (arrows) with minimal marking of interstitial pericytes (arrow heads). (B) Representative gating strategy for sorting and recovery of ZsGreen positive cells from collagenase digested renal cortices. (C) Representative electropherogram from digital gel electrophoresis demonstrating minimal sample degradation following extraction.

The present study utilized *Ren1c*Cre×Rs-ZsGreen-R (ZsGreen) mice which possess a renin-driven Cre locus. This construct mediates activation of Rosa26-promoted ZsGreen transgene resulting in constitutive ZsGreen fluorescent protein expression as a result of Lox-Stop intron excision. The above developmentally expressed transgene system resulted in consistent juxtaglomerular expression of ZsGreen with negligible expression observed in pericytes (Fig 1a). ZsGreen expressing cells constituted ~1.7% of all gated events, ~2200 ZsGreen^+/+^ cells per 1.27 x 10^5^ events, during FACS analysis of renal tissue dissociates from ZsGreen mice (Fig 1b). Total RNA recovered from these cells was of sufficient quality for microarray analysis as determined by digital electrophoresis with all samples having RNA integrity number (RIN) ≥ 8 (Fig 1c).

### Podocyte ablation perturbs global gene expression in CoRL

**Fig 2:**
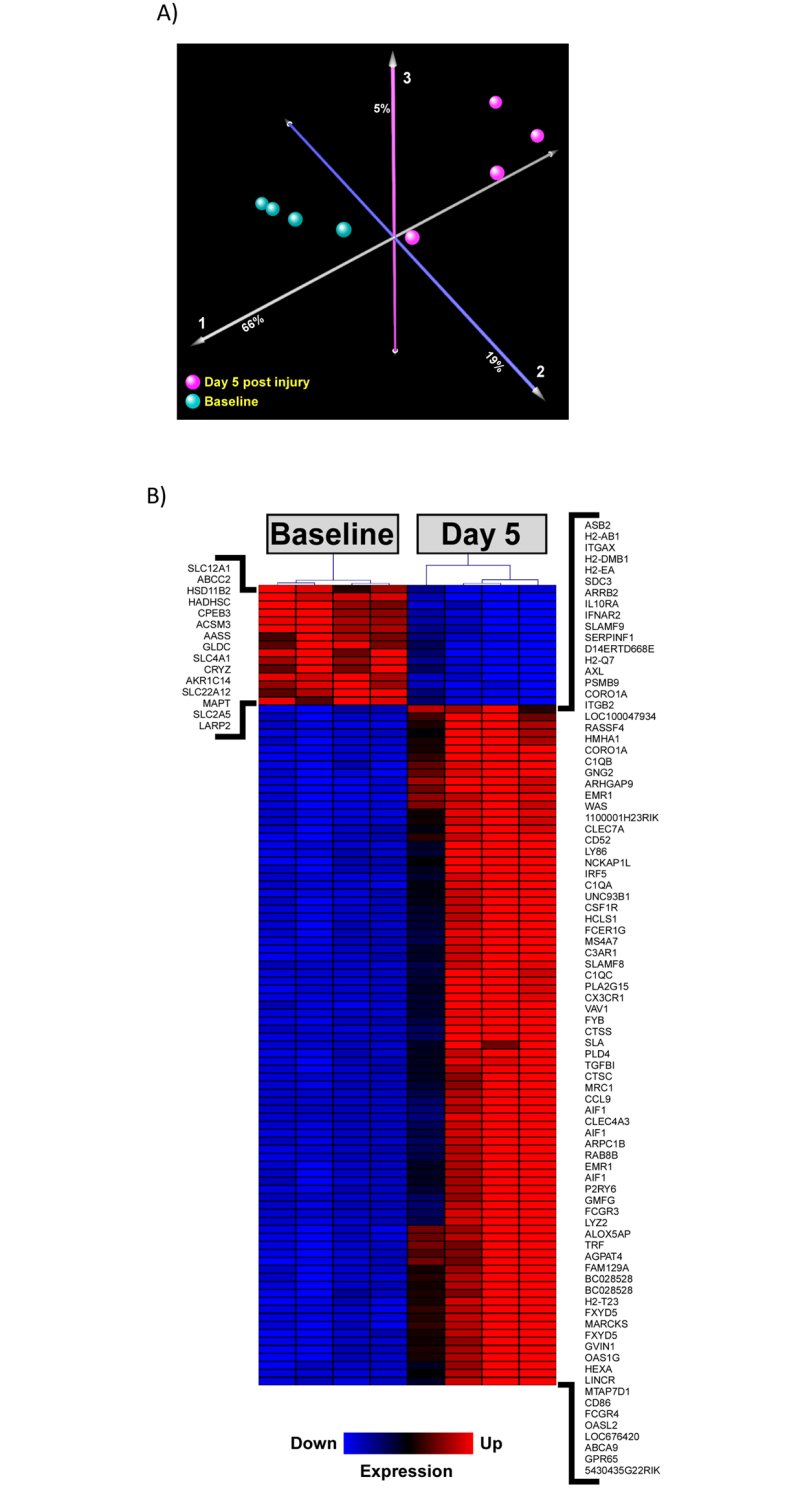
Podocyte ablation induces transcriptional changes in CoRL. (A) Principal component analysis of the entire microarray data reveals considerable changes induced in the transcriptional landscape of CoRL in response to podocyte ablation. The majority of global gene expression variability (~90%) was captured by the first three orthogonal components as depicted. (B) Heatmap representation of the top 100 most significantly dysregulated genes based on false discovery rate (FDR) analysis. Red color indicates upregulated and blue color represents downregulated expression (based on z-scores). Gene list is available in S1 Table.

Principal component analysis (PCA) aims to reduce potentially correlated high dimensional data to its linearly uncorrelated components in lower dimensional space and therefore affords simple evaluation of group-group similarity. PCA indicated the majority of variance in global gene expression between samples is captured by the first three components and that uninjured CoRL (Fig 2a, cyan spheres) segregated from day 5 post-disease induction CoRL (Fig 2a, magenta spheres). This unbiased approach implied that the primary driver of gene expression variability is significant inter-group transcriptional phenotypes and not intra-group differences.

Inter-sample variance was appropriately controlled for via variance stabilization transformation followed by quantile normalization (S1 Fig). Furthermore, intra-group similarity and inter-group variance, as indicated by PCA plot, is highlighted in heatmap representation of the top 100 most significantly dysregulated genes (ranked by FDR) where the heatmap dendrogram represents sample-to-sample similarity (Fig 2b). At the global level, more genes were observed to be downregulated than upregulated (9,966 *vs* 8,172 respectively) (S2 Fig).

### Immune and metabolic-related genes were inversely dysregulated in CoRL following podocyte ablation

**Fig 3:**
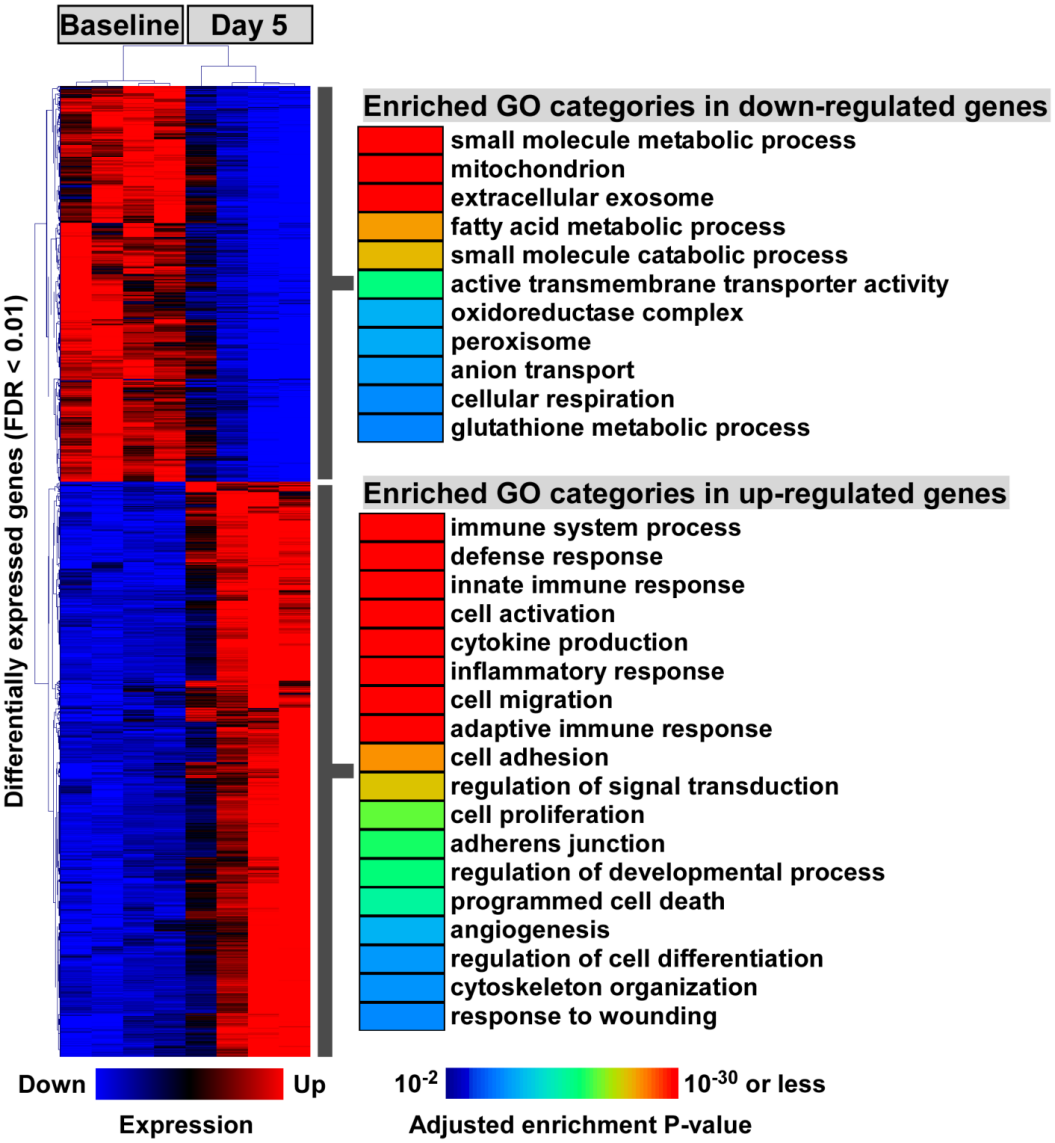
Functional enrichment of GO annotations among differentially expressed genes reveals major themes of immune, repair, and metabolism-related processes. Two dimensional hierarchical clustering of differentially expressed genes (FDR ≤ 0.01) identified two distinct expression patterns following podocyte ablation. GO terms enriched in downregulated genes (upper section) predominately cluster into various aspects of cellular metabolism. GO annotations enriched among upregulated genes (lower section) display a range of functional categories covering immune response, cell migration and cell differentiation.

To evaluate how podocyte ablation altered the functional state of CoRL, we performed Gene Ontology (GO) analysis on differentially up and downregulated genes (FDR ≤ 0.01) [26]. GO terms enriched in downregulated genes predominately clustered into metabolism related categories such as *mitochondrion*, *fatty acid metabolic process*, *oxidoreductase complex* and *cellular respiration* which may indicate decreased metabolic activity (Fig 3, S2 Table). Interestingly, *glutathione metabolic process* was also enriched among downregulated genes indicating oxidative stress may play a role in the response of CoRL to podocyte ablation.

Conversely, GO term enrichment among upregulated genes clustered into two broad classifications, namely immune-related processes and remodeling/reparative processes (Fig 3, S3 Table). Examples of the former group included *immune system process*, *cytokine production*, *inflammatory response* and *adaptive immune response* with examples of the latter including *cell activation*, *cell migration*, *cell adhesion* and *cell proliferation*. Additionally, clusters which may indicate a transdifferentiation-like phenotype, such as *regulation of developmental process* and *regulation of cell differentiation*, were also highly enriched in the upregulated genes. Collectively, these data indicated that CoRL undergo profound transcriptional alterations in response to abrupt podocyte ablation and that these changes may promote CoRL-mediated repair as well as transdifferentiation/migration.

### Podocyte ablation suppresses metabolic activity and activates immuno-modulatory processes in CoRL

**Fig 4:**
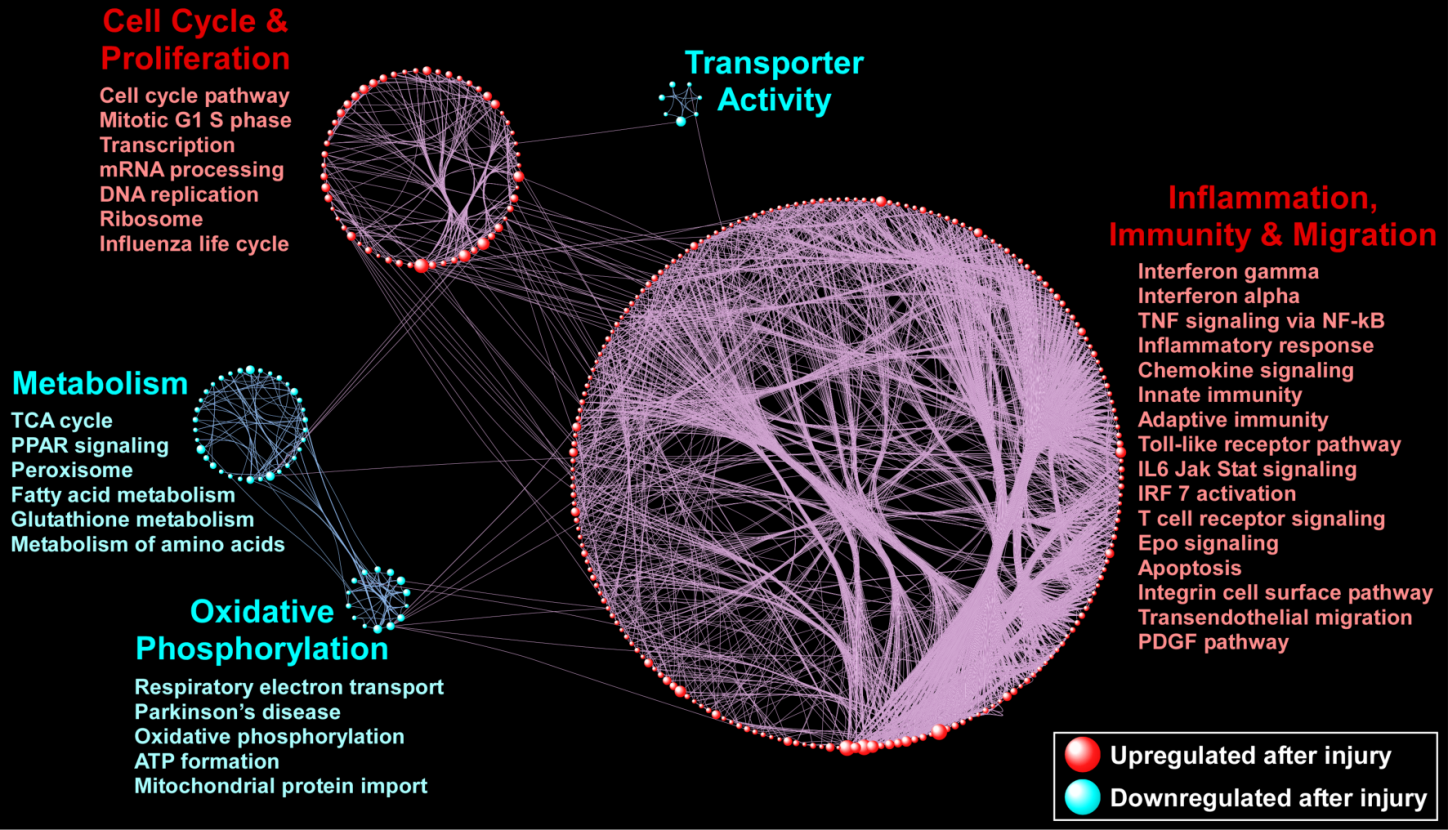
Visual summary of enriched pathways in CoRL following podocyte ablation. A gene set was considered upregulated if the expression of most of its member genes increased after injury, whereas downregulated gene sets were those with decreased gene expression. In the figure, each sphere designates an enriched pathway (red indicates upregulation and blue indicates downregulation at FDR ≤ 0.01). The size of each sphere (gene set) is proportional to the number of its members. Since pathways share many common genes, connectivity lines have been used to link these relationships and define the topology of the enrichment network. Note that gene sets with dense connections aggregate with each other due to overlap among member genes, defining larger biological “modules”. Selected gene sets have been labeled for these modules. Full list of enriched pathways is available in S4 and S5 Tables.

To gain a broader overview of pathways and processes differentially activated in CoRL following podocyte ablation, we applied Gene Set Enrichment Analysis (GSEA) to the available microarray dataset. Unlike GO, which is based on annotation of genes (and not biological pathways), and is limited to the subset of differentially expressed genes, GSEA exploits the entire transcriptome to identify enriched pathways derived from curated and canonical knowledge bases. We conducted GSEA by utilizing the combined *Hallmark* and *Canonical Pathways* datasets from MsigDB (Broad Institute; http://software.broadinstitute.org/gsea/msigdb) [27]. These databases represent well-defined biological states and curated pathways from KEGG, Reactome and Biocarta (amongst others), thereby providing a comprehensive and biologically meaningful collection of cellular processes, and signaling cascades. We identified many enriched pathways using GSEA, and applied network-based visualization to summarize the results (Fig 4).

Similar to the functional annotation clustering of GO analysis, we found that metabolism-related processes dominate the significantly downregulated gene sets (FDR ≤ 0.01). These pathways encompassed diverse metabolic processes including *oxidative phosphorylation*, *TCA cycle*, *fatty acid metabolism* and various amino acid pathways in conjunction with pathways that regulate reactive oxygen species such as *glutathione metabolism*. In contrast, GSEA revealed a large, highly interconnected module comprised primarily of upregulated immune-inflammatory pathways including interferon signaling, *TNFα signaling via NF-κB*, *IL6-JAK-STAT signaling* as well as multiple chemokine signaling pathways. Furthermore, various cell cycle, transcription, and translation pathways were also significantly upregulated in day 5 CoRL compared to baseline. Thematically, these pathways were not represented by annotation clustering of the GO results however their presence here may be linked to the *regulation of cell differentiation*, *cell activation* and *cell proliferation* GO categories. Regardless, CoRL display a clear phenotype constituting suppression of metabolic and mitochondrial processes and widespread activation of immune- and cell cycle-related pathways.

### HNF1-, ETS- and IRF-family transcription factors may drive changes in CoRL following podocyte loss

Transcription factors are a major control point for expression of any gene, and as such, determination of enrichment in transcription factor-specific gene sets may highlight which of these regulators play a major role in the observed phenotype. Applying GSEA using well-curated transcription factor gene sets, we found that several HNF-family transcription factors were downregulated in day 5 CoRL compared to baseline CoRL, suggesting that HNF-family transcription factors may play a role in the maintenance of the native CoRL phenotype (Table 1). Conversely, the CoRL phenotype induced by abrupt podocyte ablation may be mediated by ETS-family transcription factors with a number of gene sets for specific family members ranking in the 20 most significantly upregulated gene sets (Table 2, S6 Table). IRF-family of transcription factors were also highly ranked indicating that both ETS- and IRF-family transcription factors may mediate the phenotype observed in CoRL at 5 days post podocyte ablation.

**Table 1:** Transcription factor gene sets downregulated in CoRL at day 5 compared to baseline CoRL. NES: normalized enrichment score; FDR: false discovery rate.

NAME	NES	FDR
ERR1_Q2	-1.883	0.008
HNF1_Q6	-1.848	0.006
SF1_Q6	-1.804	0.007
YNTTTNNNANGCARM_UNKNOWN	-1.792	0.006
HNF1_01	-1.713	0.012
HNF4_DR1_Q3	-1.600	0.044
TTANTCA_UNKNOWN	-1.591	0.043

**Table 2:** Top 20 transcription factor gene sets upregulated in CoRL at day 5 FSGS compared to baseline. NES: normalized enrichment score; FDR: false discovery rate.

NAME	NES	FDR
ELF1_Q6	2.260	≤ 0.001
PEA3_Q6	2.109	≤ 0.001
ETS_Q4	2.089	≤ 0.001
IRF_Q6	2.059	≤ 0.001
YAATNANRNNNCAG_UNKNOWN	2.022	≤ 0.001
TTCYNRGAA_STAT5B_01	2.013	≤ 0.001
ELK1_01	1.992	≤ 0.001
ETS1_B	1.981	≤ 0.001
ETS2_B	1.966	≤ 0.001
PU1_Q6	1.914	5.41E-04
NERF_Q2	1.911	4.92E-04
GGARNTKYCCA_UNKNOWN	1.909	4.51E-04
YGTCCTTGR_UNKNOWN	1.905	4.16E-04
ISRE_01	1.878	4.63E-04
NFKAPPAB65_01	1.803	0.002
STAT3_01	1.786	0.002
NFKAPPAB_01	1.784	0.002
CREL_01	1.751	0.004
TEL2_Q6	1.744	0.005
NFKB_Q6	1.740	0.005

### Biological significance of transcription factor gene set enrichment

The biological impact of a given transcription factor was determined through integration of *transcription factor* and *canonical pathways/hallmark* gene sets. In this analysis, a search space was generated for baseline and day 5 CoRL composed of *transcription factor* and *canonical pathways/hallmark* which were significantly enriched according to GSEA testing (FDR ≤ 0.01 for downregulated gene sets, FDR ≤ 0.001 for upregulated gene sets). For each sample group, *transcription factor* gene set member genes were searched for in each *canonical pathway/hallmark* gene set. Member genes contained within each transcription factor/pathway intersection were filtered for significance (FDR ≤ 0.01 for baseline gene sets, FDR ≤ 0.001 for day 5 gene sets) and also for the direction of dysregulation (downregulation for downregulated gene sets, upregulation for upregulated gene sets). By utilizing only gene sets that were significantly enriched and removing nonsignificantly dysregulated genes, the resultant dataset captured biologically relevant interactions between genes, their host pathway and the putative transcription factors targeting them.

**Fig 5:**
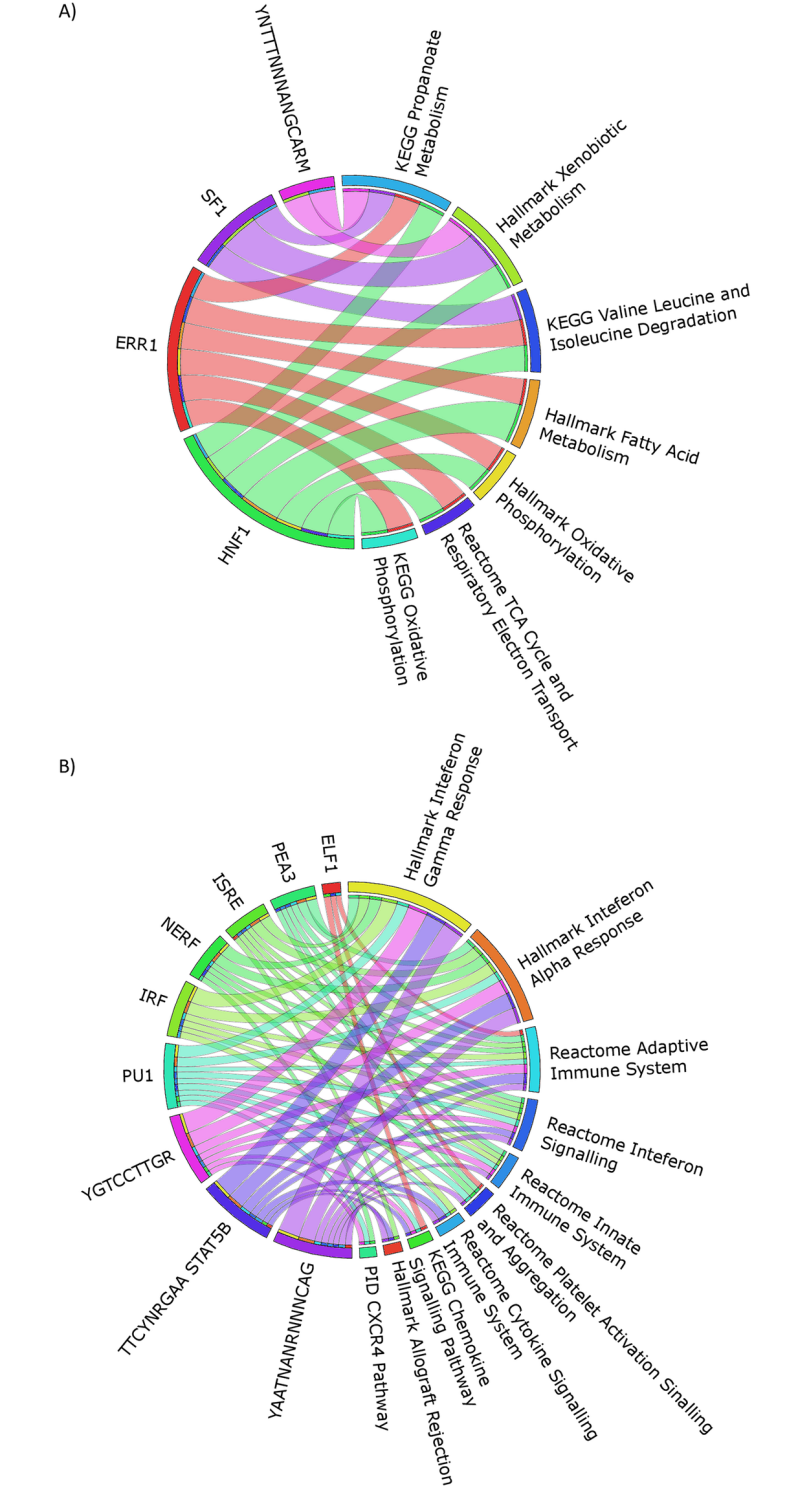
Circos plots representing interconnectivity between significantly enriched *transcription factor* and *canonical pathway/hallmark* gene sets. (A) Connection between gene sets downregulated in day 5 CoRL. Only genes and gene sets significantly downregulated (FDR ≤ 0.01) were considered. Transcription factors targeting only one pathway are not depicted. (B) Circos plot representing interconnectivity between upregulated gene sets in day 5 CoRL. Only genes and gene sets significantly upregulated (FDR ≤ 0.001) were considered. Gene set connections with fewer than four genes were discarded to afford plot simplification. Transcription factors targeting only one pathway are not depicted. Ribbon thickness represents the number of genes in the connection. The full result tables for baseline and day 5 CoRL may be found in S7 and S10 Tables respectively.

Connectivity between *transcription factor* and *canonical pathway/hallmark* gene sets were represented by Circos plots with the ribbon thickness representing the number of genes in each intersection [28]. The contingency table used to generate the plot, in addition to the intersectional gene lists for baseline intersections are available in the supplementary data **(**S7 and S8 Tables **respectively)**. In baseline CoRL, ERR1 (red ribbons) and HNF1 (green ribbons) possessed the greatest number of pathway connections and also targeted many of the same pathways (Fig 5a, S7 Table). The vast majority of pathways regulated by these transcription factors are involved in ETC and TCA-related processes (Fig 5a, S8 Table). SF1 (purple ribbons) also appears to contribute to the downregulation of metabolic processes with connections made with a number of pathways involved in metabolite and amino acid turnover (Fig 5a, S8 Table). Despite these clear associations, only ERR1 was found to be downregulated in day 5 CoRL compared to baseline and therefore, based on this analysis, is the most likely candidate for the downregulation of CoRL metabolic processes compared to either SF1 or HNF1 transcription factors.

To reduce plot complexity, intersections between *transcription factor* and *canonical pathway*/*hallmark* gene sets upregulated in day 5 CoRL were filtered for connections with at least four member genes in addition to limiting gene sets and genes whose enrichment/dysregulation was highly significant (FDR ≤ 0.001). The contingency tables used to generate the plots, in addition to the intersectional gene lists for baseline intersections are available in in the supplementary data (S10 and S11 Tables **respectively)**. Here, a poorly characterized transcription factor with a YAATNANRNNNCAG recognition motif (purple ribbons) was the most connected in regards to the number of pathways targeted and the number of member genes within those connections (Fig 5b, S10 Table). Importantly, the pathways targeted by this transcription factor are representative of the phenotype indicated by GO cluster analysis and pathway GSEA, namely upregulation of various immune-related processes, cell cycle and RNA-biology (Fig 5b, S11 Table). IRF (light green ribbons) also possessed a high degree of connectivity among the represented pathways including IFNα/ϒ pathways in addition to innate and adaptive immune system pathways (Fig 5b, S11 Table). Additionally, the PU-box transcription factor (pale blue ribbons) gene set had connections with a subset of pathways targeted by either the YAATNANRNNNCAG motif transcription factor or IRF (Fig 5b, S10 and S11 Tables). Both IRF1 and the PU-box family member SPIC are significantly upregulated in day 5 CoRL compared to baseline CoRL and may therefore be major contributors to the upregulation of immune-related processes.

### Quantitative real-time polymerase chain reaction validation of microarray analysis

**Fig 6:**
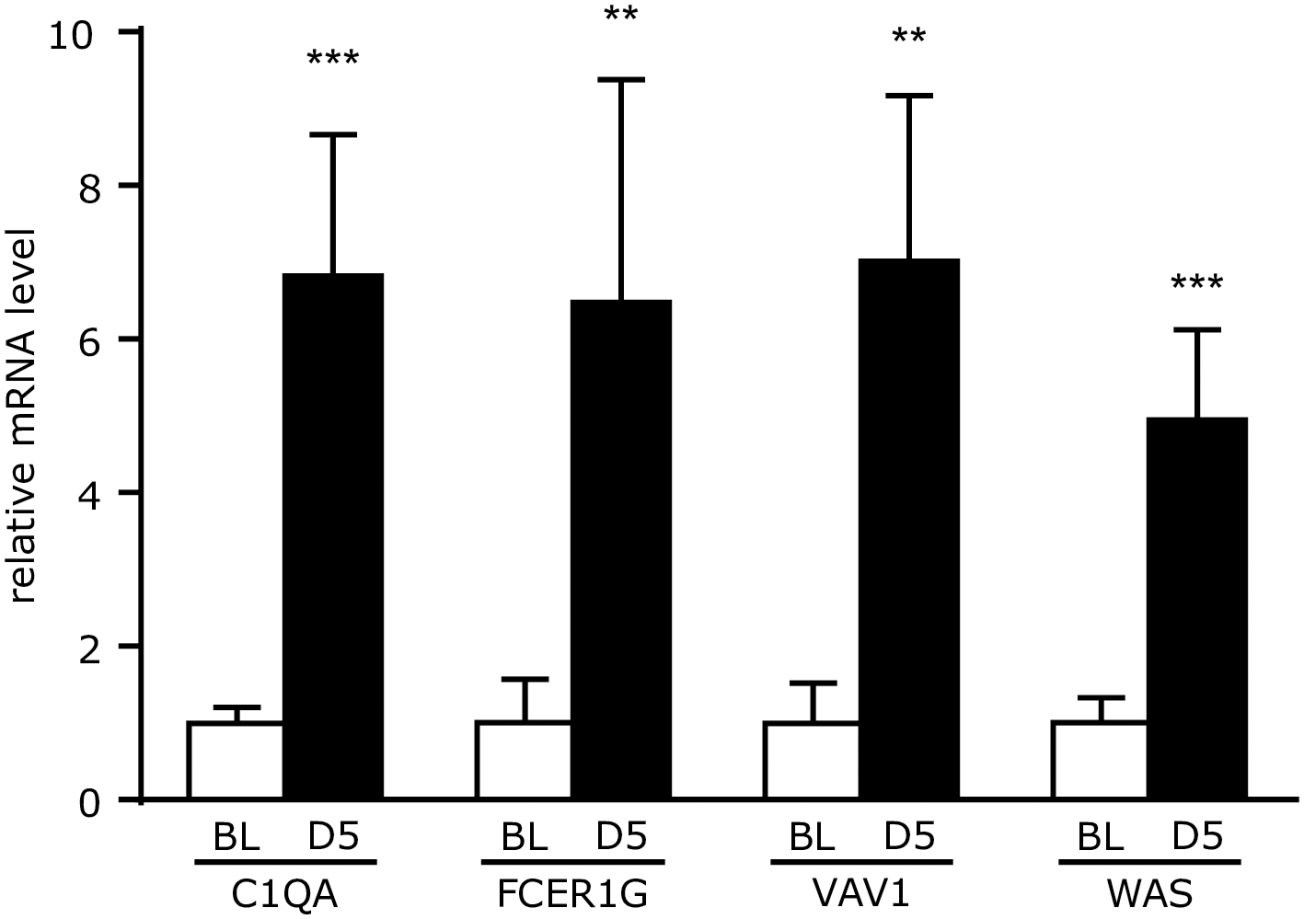
Confirmation of gene expression changes returned from integrated analysis. Expression levels for the top 5 genes returned from the integrative analysis are, as in the microarray, significantly upregulated.

From the integrated analysis of transcription factor and canonical pathway gene sets, the top 4 most upregulated genes were chosen for validation of the microarray data by qRT-PCR. These genes, namely, Complement C1q A Chain (C1QA), Fc Fragment Of IgE Receptor Ig (FCER1G) Vav Guanine Nucleotide Exchange Factor 1 (VAV1) and Wiskott-Aldrich Syndrome (WAS) were all confirmed to be significantly upregulated (Fig 6).

## Discussion

Cells of renin lineage (CoRL) are unique kidney cells which are an essential part of the juxtaglomerular apparatus and the renin-angiotensin system [29]. CoRL have recently garnered much interest for their propensity to act as adult podocyte progenitors following podocyte loss [8–10, 30, 31]. To better understand potential mechanisms and pathways, we present an unbiased bioinformatics overview of the CoRL transcriptome following abrupt podocyte depletion.

By day 5 of experimentally-induced FSGS, widespread differential gene expression was observed in FACS isolated CoRL. Functional and gene set enrichment analysis revealed that up and downregulated CoRL genes mapped to highly distinct processes, indicating selective activation and suppression of pathways in response of podocyte depletion. We confirmed the upregulation of several genes by qPCR as a means to validate the microarray data. All validated genes were targets of the ELF1 transcription factor whose gene set was the most significantly upregulated in day 5 CoRL. Importantly, ELF1 gene set member genes associated with pathways regulating the actin cytoskeleton, chemokine signaling and focal adhesion; all of which are important for cellular migration and transdifferentiation [32–35]. It is therefore tempting to speculate that ELF1 may mediate CoRL-to-podocyte transdifferentiation through regulation of a core set of genes targeting the aforementioned pathways.

Conversely, the most significantly downregulated genes of negatively enriched *transcription factor* and *canonical pathway* gene sets were expectedly involved in some aspect of catabolic or anabolic metabolism including a number of ATPase subunits. This is further supported by association of ERR1 transcription factor with the top ranking genes as this transcription factor has reported roles in various metabolic processes [36, 37]. Furthermore, pathways possessing the greatest number of ERR1 targets were either mitochondria-specific or define pathologies which are thought to be predominately mitochondrial in nature such as Parkinson’s and Alzheimer’s diseases [38]. Interestingly, peroxisome and glutathione pathways were also negatively enriched (FDR ≤ 0.05) and further indicate that, at least at day 5 post podocyte ablation, CoRL no longer maintain normal metabolic functions [39–41]. GO analysis reflected this, albeit in more general terms, with processes pertaining to carboxylic acid catabolism, organic acid catabolism, cellular respiration, amino acid catabolism and ETC all occupying the top ranked positions.

In addition to the aforementioned metabolic pathways various RNA-related pathways including *3’ UTR mediated translational regulation*, *ribosome assembly* pathway, *translation* pathway and *metabolism of RNA* were also upregulated. Enrichment of these RNA-related pathways may indicate increased protein synthesis—a critical requirement for the preparative phases of cellular transdifferentiation [42]. The absence of these processes in the integrative transcription factor/pathway analysis does not indicate they are false positives. Rather, it is likely the result of a failure to locate significantly dysregulated genes in a given *canonical pathway* that was also a member of a given *transcription factor* gene set. That is to say, no significantly enriched *transcription factor* gene sets were found to be associated with significantly dysregulated genes in any of the aforementioned RNA-related pathways.

Collectively, the phenotype of downregulated metabolic processes and upregulated inflammation-, cell cycle- and RNA-related pathways supports the notion that following podocyte loss, CoRL may serve as a podocyte progenitor sink. The triggering, maintenance and regulation of CoRL transdifferentiation and migration likely parallels that of other cellular transdifferentiation and migration events, such as occurs during epithelial wound healing [43]. One notable difference is that the immune signaling triggered in CoRL by podocyte ablation does not necessarily result in recruitment of macrophage and other leukocytes to clear wound debris. At day 14 post podocyte ablation, we have previously shown that there is no change in recruitment of macrophages, B cells, neutrophils or activated T cells to the tuft [14].

Furthermore, as CoRL reside in the juxtaglomerular apparatus anatomically upstream of the glomerular tuft, it is unlikely that the inflammatory phenotype is a direct result of CoRL insult. Rather, these changes may be triggered in a paracrine manner via the macula densa [44] or as a retrograde signal transmitted from the microvascular endothelium [45] or the tuft mesangium [46] and may serve to trigger the migration and transdifferentiation process. Regardless, it is likely that the signal is either initiated by denuded microvasculature (in both paracrine and retrograde signaling postulates) or by secretion of signaling molecules from PTC in response to changes in urinary filtrate composition (which act in a paracrine manner via the macula densa) [47].

Decreased metabolic activity may be linked to CoRL transdifferentiation potential as stem cells are known to rely more upon glycolysis than oxidative phosphorylation for metabolic output with stemness decreasing as oxidative phosphorylation increasingly becomes the major source of ATP [48]. However, glucose transport and gluconeogenesis pathways were downregulated indicating the observed suppression of mitochondrial metabolic processes possibly extend to those concerned with the utilization and generation of glucose respectively. Despite this, mTOR, which is a major regulator of mitochondrial metabolism [49] is downregulated by ~35%, which may influence transdifferentiation as is the case in ESCs [50]. In our model, the expression of several members of the peroxisome proliferator-activated receptor (Ppar) family—master regulators of metabolism—were significantly reduced, including *Pparg* (FDR < 0.05), *Ppargc1a* (FDR < 0.001), and *Ppara* (FDR < 0.05). Singhal *et*. *al*. recently demonstrated that *Pparg* expression is downregulated in podocytes from vitamin D receptor (*Vdr*) null mice via a mechanism involving suppression of sirtuin 1 (*Sirt1*) [51]. While we did not observe downregulation of *Sirt1* in our model, *Vdr* was significantly suppressed in CoRL at day 5 post-FSGS induction (FDR < 0.01). Taken together, these data suggest that dysregulation of the *Vdr*-*Pparg* network may be important in the observed suppression of metabolic pathways in CoRL following injury.

At present, there are no published methods for maintaining CoRL *in vitro*. This limits validation of the current *in vivo* findings using more targeted and mechanistic *in vitro* studies. We acknowledge that further investigation of these data and their analysis is required to gain a better understanding of the events that lead to, and mediate, the transdifferentiation and migration of CoRL following podocyte loss. We also acknowledge that this study might benefit from additional time points, although our previous studies have demonstrated that podocyte depletion with concordant microalbuminuria is well established at day 7 in this model with microalbuminuria approaching baseline levels by day 14 [14]. Furthermore, we have previously shown that migration of CoRL to the tuft occurs by day 7 post disease induction [9]. Here, day 5 may be thought of as an ‘early response time point’ where the pathological features of the model are still being established and CoRL are most active in their transcriptional response to abrupt podocyte depletion. Finally, our unbiased approach, while being comprehensive, does not identify which of the up- or down-regulated pathways play the predominant role in CoRL response to injury. Future mechanistic experiments are needed to address this important limitation.

## Conclusions

In summary, the transcriptional profile of CoRL at day 5 post-podocyte ablation indicates that these cells undergo widespread changes in cellular metabolic profile, inflammatory signaling, cytoskeletal regulation and RNA-processing. Although our findings support the model of CoRL-to-podocyte transdifferentiation, much work is still required to understand the programs that maintain CoRL plasticity and the signals that trigger their migration and differentiation.

## Supporting information

S1 Fig Sample variance boxplot and DGE summary venn diagrams. (A) Variance stabilization transformation followed by quantile normalization appropriately controls for inter-sample variance. (B) Venn diagram displaying the percentage of all genes that are either significantly or non-significantly downregulated at FDR ≤ 0.05. (C) Venn diagram displaying the percentage of all genes that are either significantly or non-significantly upregulated at FDR ≤ 0.05.
(TIF)

S1 Table Top 100 most significantly differentially expressed genes as depicted in heatmap (Fig 2b). (XLSX)

S2 Table Top 100 GO annotation clusters enriched among differentially downregulated genes in CoRL following podocyte ablation. (XLSX)

S3 Table Top 100 GO annotation clusters enriched among differentially upregulated genes in CoRL following podocyte ablation. (XLSX)

S4 Table Downregulated pathways in CoRL following podocyte ablation. (XLSX)

S5 Table Upregulated pathways in CoRL following podocyte ablation. (XLSX)

S6 Table Upregulated transcription factor gene sets in CoRL following podocyte ablation. (XLSX)

S7 Table Contingency table for Circos plot of the intersection of downregulated pathway and transcription factors. These data have been filtered according to methods.
(XLSX)

S8 Table Downregulated genes (FDR ≤ 0.01) contained within downregulated pathways and transcription factor gene sets (FDR ≤ 0.01). (XLSX)

S9 Table Fold change (log_2_) and adjusted p-values of downregulated genes which exist in both downregulated transcription factor and pathway gene sets. (XLSX)

S10 Table Contingency table for Circos plot of the intersection of upregulated pathways and transcription factors. These data have been filtered according to methods.
(XLSX)

S11 Table Upregulated genes (FDR ≤ 0.001) contained within upregulated pathways and transcription factor gene sets (FDR ≤ 0.001). (XLSX)

S12 Table Fold change (log_2_) and adjusted p-values of upregulated genes which exist in both upregulated transcription factor and pathway gene sets. (XLSX)
