# Supplementary figures and images for: Charting the transcriptional landscape of cells of renin lineage following podocyte depletion
Source: PLoS One. 2017 Dec 12;12(12):e0189084. doi: 10.1371/journal.pone.0189084 (PMC5726629; doi:10.1371/journal.pone.0189084)

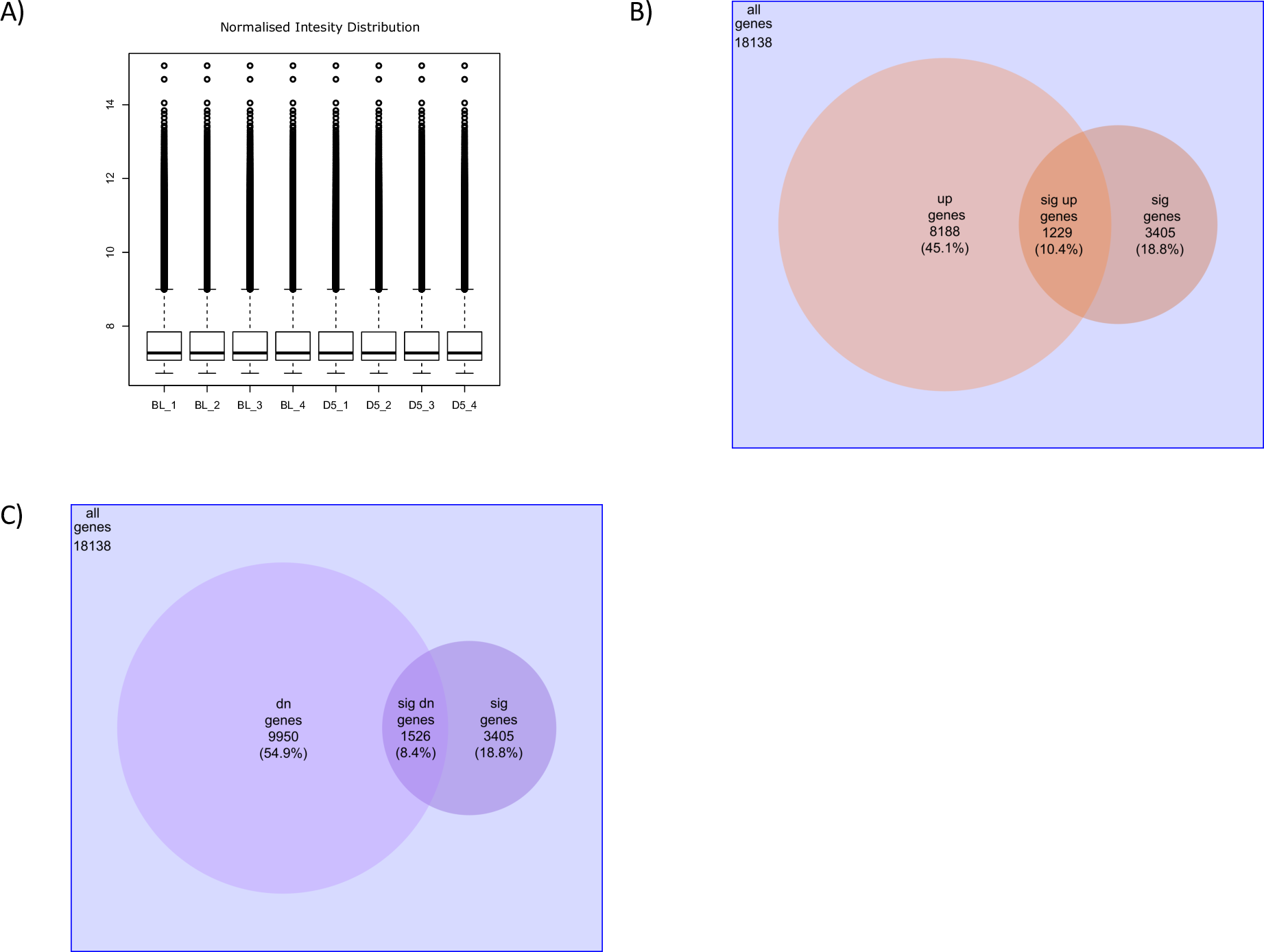

Supplement: S1 Fig — (A) Variance stabilization transformation followed by quantile normalization appropriately controls for inter-sample variance. (B) Venn diagram displaying the percentage of all genes that are either significantly or non-significantly downregulated at FDR ≤ 0.05. (C) Venn diagram displaying the percentage of all genes that are either significantly or non-significantly upregulated at FDR ≤ 0.05. (TIF) [file pone.0189084.s001.tif]
